# Bounds on Mixed State Entanglement

**DOI:** 10.3390/e22010062

**Published:** 2020-01-01

**Authors:** Bruno Leggio, Anna Napoli, Hiromichi Nakazato, Antonino Messina

**Affiliations:** 1 Laboratoire Reproduction et Développement des Plantes, Université de Lyon, ENS de Lyon, UCB Lyon 1, CNRS, INRA, Inria, 69342 Lyon, France; bruco.leggio@gmail.com; 2 Dipartimento di Fisica e Chimica-Emilio Segrè, Università degli Studi di Palermo, Via Archirafi 36, I-90123 Palermo, Italy; 3 I.N.F.N. Sezione di Catania, Via Santa Sofia 64, I-95123 Catania, Italy; antonino.messina@unipa.it; 4 Department of Physics, Waseda University, Tokyo 169-8555, Japan; hiromici@waseda.jp; 5 Dipartimento di Matematica ed Informatica, Università degli Studi di Palermo, Via Archirafi, 34, I-90123 Palermo, Italy

**Keywords:** entanglement, negativity

## Abstract

In the general framework of $d_{1}\times d_{2}$ mixed states, we derive an explicit bound for bipartite negative partial transpose (NPT) entanglement based on the mixedness characterization of the physical system. The derived result is very general, being based only on the assumption of finite dimensionality. In addition, it turns out to be of experimental interest since some purity-measuring protocols are known. Exploiting the bound in the particular case of thermal entanglement, a way to connect thermodynamic features to the monogamy of quantum correlations is suggested, and some recent results on the subject are given a physically clear explanation.

## 1. Introduction

In dealing with mixed states of a physical system, one has to be careful when speaking about entanglement. The definition of bipartite mixed state entanglement is unique (although problems may arise in dealing with multipartite entanglement [1]), but its quantification relies on several different criteria and it is not yet fully developed: many difficulties have arisen in the definition of physically sensible measures [2,3]. The main problem affecting a few known mixed state entanglement measures is indeed the fact that extending a measure from a pure state case to a mixed state case usually requires challenging maximization procedures over all its possible pure state decompositions [4,5,6]. Notwithstanding, the investigation of the connection between entanglement and mixedness exhibited by a quantum system is of great interest, for example, in quantum computation theory [7,8] and in quantum teleportation [9]. The threshold of mixedness exhibited by a quantum system compatible with the occurrence of entanglement between two parties of the same system has been analyzed, leading for example to the so-called Kus-Zyczkowski ball of absolutely separable states [10,11,12,13]. Quite recently, possible links between entanglement and easily measurable observables have been exploited to define experimental protocols aimed at measuring quantum correlations [14,15,16]. The use of measurable quantities as entanglement witnesses for a wide class of systems has been known for some time [17,18], but an analogous possibility amounting at entanglement measures is a recent and growing challenge. To the present day, some bounds for entanglement are measured in terms of correlation functions in spin systems [19] or using quantum quenches [20]. Indeed, an experimental measure of entanglement is, generally speaking, out of reach because of the difficulty in addressing the local properties of many-particle systems and of the fundamental non-linearity of entanglement quantifiers. For this reason, the best one can do is to provide experimentally accessible bounds on some entanglement quantifiers [21]. The aim of this paper is to build a bound to the entanglement degree in a general bipartition of a physical system in a mixed state. We are going to establish an upper bound to the negativity *N* [22] in terms of the linear entropy $S_{L}$. We are thus studying what is called negative partial transpose (NPT) entanglement. It should however be emphasized that a non-zero negativity is a sufficient but not necessary condition to detect entanglement, since positive partial transpose (PPT, or bound) entanglement exists across bipartitions of dimensions higher than 2×3, which cannot be detected by means of the negativity criterion [23]. Our investigation contributes to the topical debate concerning a link between quantum correlations and mixedness [24]. We stress that our result is of experimental interest since the bound on *N* may easily be evaluated by measuring the linear entropy.

## 2. An Upper Bound to the Negativity in Terms of Linear Entropy

Consider a *d*-dimensional system S in a state described by the density matrix $\left(0\;\leq p_{i}\;\leq \;1,\;\;\;\forall i\right)$

(1) $$\rho =\sum_{i}p_{i}\sigma_{i},$$

where each $\sigma_{i}$ represents a pure state, and define a bipartition into two subsystems S${}_{1}$ and S${}_{2}$ with dimensions $d_{1}$ and $d_{2}$ respectively ($d=d_{1}\cdot d_{2}$). It is common [19] to define negativity as

(2) $$N=\frac{∥\rho^{T_{1}}∥-1}{d_{m}-1}=\frac{\mathrm{Tr}\sqrt{\rho^{T_{1}}{\left(\rho^{T_{1}}\right)}^{†}}-1}{d_{m}-1},$$

where $d_{m}=\min\left\{d_{1},d_{2}\right\}$, $\rho^{T_{1}}$ is the matrix obtained through a partial transposition with respect to the subsystem $S_{1}$ and ∥·∥ is the trace norm ($∥O∥\equiv \mathrm{Tr}\{\sqrt{OO^{†}}\}$). In what follows we will call $d_{M}=\max\left\{d_{1},d_{2}\right\}$. By construction, 0≤N≤1, with N=1 for maximally entangled states only. Furthermore, the linear entropy $S_{L}$ in our system is defined as

(3) $$S_{L}=\frac{d}{d-1}\left(1-\mathrm{Tr}\rho^{2}\right)=\frac{d}{d-1}P_{E},$$

where $P_{E}=1-\mathrm{Tr}\rho^{2}=1-{∥\rho ∥}_{2}^{2}$ is a measure of mixedness in terms of the purity $\mathrm{Tr}\rho^{2}$ of the state, ${∥\rho ∥}_{2}$ being the Hilbert–Schmidt norm of ρ (${∥O∥}_{2}\equiv \sqrt{\mathrm{Tr}\{OO^{†}}\}$). By definition, $S_{L}=0$ for any pure state while $S_{L}=1$ for maximally mixed states. It is easy to see that there exists a link between the trace norm of an operator *O* in a *d*-dimensional Hilbert space and its Hilbert–Schmidt norm. Such a link can be expressed as

(4) $${∥O∥}^{2}=(\sum_{i=1}^{d}|\lambda_{i}{\left|\right)}^{2}\leq d\sum_{i=1}^{d}|\lambda_{i}{|}^{2}=d{∥O∥}_{2}^{2},$$

where $\lambda_{i}$ is the *i*-th eigenvalue of *O* and the so-called Chebyshev sum inequality ${\left(\sum_{i=1}^{d}a_{i}\right)}^{2}\leq d\sum_{i=1}^{d}a_{i}^{2}$ has been used. Since, in addition, the Hilbert–Schmidt norm is invariant under partial transposition, one readily gets a first explicit link between negativity and mixedness $P_{E}$, valid for generic *d*-dimensional systems, in the form of an upper bound, which reads

(5) $$N\leq \frac{\sqrt{d}\sqrt{1-P_{E}}-1}{d_{m}-1}\equiv Q_{1}.$$

Equation (5) provides an upper bound to the negativity *N* in terms of $P_{E}$ and thus, in view of Equation (3), in terms of the linear entropy. This bound imposes a maximal zero value for *N* only for a maximally mixed state, the maximum being taken over all bipartite quantum states of the same purity. It is known [10,11], however, that no quantum state of *S* can exhibit bipartite entanglement if its purity is smaller than or equal to ${\left(d-1\right)}^{-1}$. In other words, the noise due to the mixedness of the state is too high for bipartite correlations to exist. Additionally, in the case of a pure (or almost pure) state, the bound becomes useless as long as the bipartition is not “balanced” (by “balanced” we mean a bipartition where $d_{m}=\sqrt{d}$). It indeed becomes greater than one (thus being unable to give information about entanglement) for mixedness smaller than $\frac{d\;-\;d_{m}^{2}}{d}$, which might even approach 1 in some specific cases (recall that, by definition, $d_{m}\leq d$). We however expect entanglement to be unbounded only in the case of pure states ($P_{E}=0$). In the following we show that bound (5) can be strengthened.

## 3. Strengthening the Previous Bound

Observe firstly that the rank $r_{\rho }$ of $\rho^{T_{1}}$ is not greater than $d_{m}^{2}$ (equal to *d*) when ρ is pure (maximally mixed). For this reason we write

(6) $$r\left(S_{L}\right)\equiv \max_{\left\{\rho :\mathrm{Tr}\rho^{2}=1-\frac{d-1}{d}S_{L}\right\}}r_{\rho }.$$

By construction, $r\left(0\right)=d_{m}^{2}$ since any pure state can be written in Schmidt decomposition consisting of $d_{m}$ vectors, and r(1)=d because a maximally mixed state is proportional to identity. Since by definition ${\left(\sum_{i=1}^{d}\left|\lambda_{i}\right|\right)}^{2}={\left(\sum_{i=1}^{r(S_{L})}|\lambda_{i}|\right)}^{2}$ holds for any physical system, Equation (5) may be substituted by the following inequality:

(7) $$N\leq \frac{\sqrt{r(S_{L})}\sqrt{1-P_{E}}-1}{d_{m}-1}.$$

Note however that there are at least some physical systems for which the function in (6), due to the maximization procedure involved in its definition, is always equal to *d* in the range $S_{L}\in \left(0,1\right]$, showing then a discontinuity at $S_{L}=0$ as

(8) $$\lim_{S_{L}\to 0}r\left(S_{L}\right)=d\neq d_{m}^{2}=r\left(0\right).$$

Since we want our result to hold generally, independently of the particular system analyzed, Equation (7) cannot improve Equation (5) because even for slightly mixed states $\left(0<S_{L}<<1\right)$ we have a priori no information on $r(S_{L})$ which might be equal to *d*, tracing back Equation (7) to Equation (5). Despite this, we may correct (7) exploiting the expectation that for very low mixedness some of these eigenvalues are much smaller than the others. Indeed, for all the $r(S_{L})$, non-vanishing eigenvalues appearing in Equation (4) are treated on equal footing in going from $∥\rho^{T_{1}}∥$ to $∥\rho^{T_{1}}{∥}_{2}$. To properly take into account the difference between them, go back to Equation (1) and define a reference pure state $\sigma_{R}$ at will among the ones having the largest occupation probability $p_{R}$. The spectrum of $\sigma_{R}^{T_{1}}$ consists of $n_{p}$ non-zero eigenvalues $\left\{\mu_{\alpha }^{\left(R\right)}\right\}$ $\left(\max n_{p}=d_{m}^{2}\right)$ and of $n_{m}=d-n_{p}$ zero eigenvalues $\left\{\nu_{\beta }^{\left(R\right)}\right\}$.

We call the former α-class eigenvalues and the latter β-class eigenvalues, and obviously the latter class does not contribute to $∥\sigma_{R}^{T_{1}}∥$. In order to strengthen (5) we are interested in the spectrum of $\rho^{T_{1}}$ which generally consists of *d* non-zero eigenvalues. Unfortunately, then, we cannot directly introduce analogous α- and β-classes to identify which eigenvalues contribute to the sum involved in Equation (4) comparatively much less than the other ones, when the state ρ possesses a low mixedness degree and is thus very close to a pure state. To overcome this difficulty, let us consider a parameter-dependent class of density matrices associated to the given ρ

(9) $$\tau \left(x\right)=\sum_{i}q_{i}\left(x\right)\sigma_{i}$$

with 0≤x≤1, such that $\tau \left(0\right)=\sigma_{R}$ and τ(1)=ρ. This means that, for all *i*,

(10) $$\begin{array}{ccc} \lim_{x\to 0}q_{i}\left(x\right) & = & \delta_{iR}, \end{array}$$

(11) $$\begin{array}{ccc} \lim_{x\to 1}q_{i}\left(x\right) & = & p_{i}. \end{array}$$

In addition, we assume that all $q_{i}\left(x\right)$ are continuous functions of *x*. Thus, $\tau {\left(x\right)}^{T_{1}}$ continuously connects $\rho^{T_{1}}$ and $\sigma_{R}^{T_{1}}$ and, as a consequence, any $\nu_{\beta }^{\left(R\right)}$ is continuously connected to a particular eigenvalue of $\rho^{T_{1}}$, which will be the the corresponding mixed state β-class eigenvalue $\nu_{\beta }$. In this way one can define the function $\nu_{\beta_{0}}\left(x\right)$ as the eigenvalue of $\tau {\left(x\right)}^{T_{1}}$ having the property

(12) $$\lim_{x\to 0}\nu_{\beta_{0}}\left(x\right)=\nu_{\beta_{0}}^{\left(R\right)}$$

and so the β-class eigenvalue for $\rho^{T_{1}}$ as

(13) $$\nu_{\beta_{0}}\equiv \lim_{x\to 1}\nu_{\beta_{0}}\left(x\right).$$

We emphasize at this point that the results of this paper do not depend on the explicit functional dependence of τ(x) on *x*, which can be chosen at will provided it satisfies conditions (11), nor on the range of variability of *x* itself. Indeed, τ(x) and *x* are just mathematical tools, with (in general) no physical meaning. To save some writing and in view of Equation (4), we put

(14) $$A=\sum_{\alpha }\mu_{\alpha }^{2}\;\;\;\;B=\sum_{\beta }\nu_{\beta }^{2}$$

and notice that $\mathrm{Tr}{\left(\rho^{T_{1}}\right)}^{2}=\mathrm{Tr}\rho^{2}=A+B$. We can now state (see Appendix A for a proof) the following.

**Lemma** **1.** *Given a state ρ of a system in a d-dimensional Hilbert space, and the associated reference pure state $\sigma_{R}$, for any set of states τ(t) satisfying (9) and (11) there exists a value δ≥1 such that 1−A(t)−B(t)≥B(t)d for any t∈[0,δ]. This result is of course based on the possibility to define a reference pure state $\sigma_{R}$. As previously explained, such a possibility rests on the assumed existence of one (or a subset of) $p_{i}$ in one pure-state decomposition of ρ, such that $p_{i}>p_{j},j\neq i$. In other words, one (or a subset of) pure state(s) must be occupied with probability higher (and not equal to) the populations of the rest of the pure states. If this is verified, then the reference pure state $\sigma_{R}$ is well defined.*

Lemma 1 allows us to find a function $w(S_{L})$ such that $w\left(0\right)=d_{m}^{2}$ and

(15) $$∥\rho^{T_{1}}{∥}^{2}\leq w\left(S_{L}\right)=f(∥\rho^{T_{1}}{∥}_{2}^{2}).$$

Starting from the identity

(16) $$\begin{array}{c} ∥\rho^{T_{1}}{∥}^{2}=(\sum_{\alpha }^{d_{m}^{2}}|\mu_{\alpha }{\left|\right)}^{2}+(\sum_{\beta }^{d-1}|\nu_{\beta }{\left|\right)}^{2}+2\sum_{\alpha }^{d_{m}^{2}}|\mu_{\alpha }|\sum_{\beta }^{d-1}\left|\nu_{\beta }\right| \end{array}$$

and applying the Chebyshev sum inequality term-by-term, we obtain

(17) $$∥\rho^{T_{1}}{∥}^{2}\leq {\left(d_{m}\sqrt{A+B}+\sqrt{\frac{d-1}{d}}\sqrt{1-A-B}\right)}^{2},$$

where Lemma 1 has been exploited. Expressing Equation (17) in terms of negativity and purity, we finally get

(18) $$N\leq \frac{d_{m}\sqrt{1-P_{E}}+\sqrt{\frac{d-1}{d}}\sqrt{P_{E}}-1}{d_{m}-1}\equiv Q_{2}.$$

Bound (18) improves bound (7) for high purity when $S_{L}$ is small (i.e., $Q_{2}<Q_{1}$), generally becoming greater than $Q_{1}$ at low purity. In addition, it still suffers the same drawback as $Q_{1}$, not vanishing when $1-P_{E}=\frac{1}{d\;-\;1}$. In such a case one has to consider the lower bound $\frac{1}{d\;-\;1}$ on purity, below which no entanglement survives. In order to take such a bound into account, instead of distinguishing among α and β eigenvalues of $\rho^{T_{1}}$, we can divide them into non-negative ones $\left\{\xi_{i}\right\}$ and negative ones $\left\{\chi_{i}\right\}$. In this way, calling $n_{-}$ and $\left(d-n_{-}\right)$ the numbers of negative and non-negative eigenvalues, respectively, and applying the Lagrange multiplier method to the function $∥\rho^{T_{1}}∥=\sum_{i}^{d-n_{-}}\xi_{i}+\sum_{j}^{n_{-}}\chi_{j}$ subjected to constraints $\sum_{i}\xi_{i}^{2}+\sum_{j}\chi_{j}^{2}=1-P_{E}$ and $\sum_{i}\xi_{i}+\sum_{j}\chi_{j}=1$, one finds

(19) $$∥\rho^{T_{1}}{∥}^{2}\leq \frac{d-2n_{-}+2\sqrt{n_{-}\left(d-n_{-}\right)\left(d\left(1-P_{E}\right)-1\right)}}{d}.$$

Bound (19) can be exploited to show that no entanglement can survive at purity lower than $\frac{1}{d\;-\;1}$. Indeed, for entanglement to exist, at least one eigenvalue has to be negative. However, by normalization, it always has to be true that $∥\rho^{T_{1}}∥\geq 1$ and this implies that, as long as $n_{-}\geq 1$, purity $1-P_{E}$ cannot be smaller than $\frac{1}{d-1}$ as expected. However, in general, the number of negative eigenvalues is not known. In these cases, the best one can do is to look for the maximum, with respect to $n_{-}$, of the right-hand side of (19), leading unfortunately once again to bound (5) on *N*. However, since always $N\leq \Theta \left(\frac{d-2}{d-1}-P_{E}\right)\equiv Q_{3}$, where Θ(x) is the Heaviside step function, defining $Q=\min\{Q_{1},Q_{2},Q_{3}\}$, we state our final and main result as

(20) N≤Q,

valid for every possible bipartition of a quantum system, independent of its (finite) dimension, its detailed structure, or its properties. It is worth stressing that computing negativity quickly becomes a very hard task as the dimension of the Hilbert space grows, while the evaluation of purity can be performed without particular efforts. We emphasize in addition that bound *Q* in Equation (20) only depends on purity, and is completely determined once a bipartition of the physical system is fixed and purity is known. This means that an experimental measure of purity allows the extraction of information about the maximal degree of bipartite entanglement one can find in the system under scrutiny. Some purity-measuring protocols, or at least purity estimations based on experimental data, have been proposed. They are based on statistical analysis of homodyne distributions, obtained measuring radiation field tomograms [25], on the properties of graph states [26], or on the availability of many different copies of the state over which separable measurements are performed [27]. In all cases where a measure of purity is possible, an experimental estimation of bipartite entanglement is available thanks to Equation (20), which is then actually experimentally accessible.

## 4. Crossover between $Q_{1}$ and $Q_{2}$ and Numerical Results

As commented previously, bounds $Q_{1}$ (Equation (5)) and $Q_{2}$ (Equation (18)) can supply information about bipartite entanglement in two different setups. Indeed, $Q_{1}$ is accurate enough for a balanced bipartition (i.e., when $d_{m}∼\sqrt{d}$) but fails when $\sqrt{d}\gg d_{m}$ since it rapidly becomes greater than 1. To solve this problem, we obtained the bound $Q_{2}$ which, by construction, provides nontrivial information about bipartite entanglement in an unbalanced bipartition $\left(\sqrt{d}\gg d_{m}\right)$, but may not work properly for a balanced one. In order to build the last bound $Q_{2}$ in the previous section we started with a more detailed analysis of the structure of the eigenvalues after partial transposition. This analysis leads us, specifically, to study the rank of the partial transpose, as this is one key parameter used in the Chebyshev inequality. In this section we demonstrate the existence of a special regime of the purity wherein the helpfulness of $Q_{2}$ against $Q_{1}$ emerges in a very transparent way. It is actually a very easy task to show that our new bound $Q_{2}$ works better than the old one, $Q_{1}$ (i.e., $Q_{1}\geq Q_{2}$), when the purity *P* is greater than a critical value given in terms of the total Hilbert space dimension and of the subdimensions of the bipartition—that is, when

(21) $$P\left(\rho \right)\geq \frac{d-1}{d{\left(\sqrt{d}-d_{m}\right)}^{2}+d-1}=P_{c}.$$

**Figure 1:**
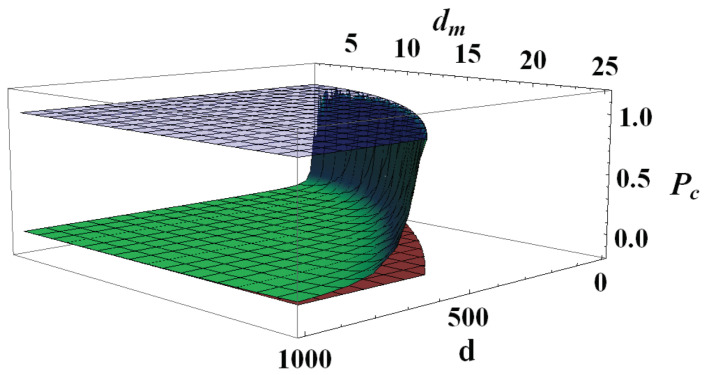
(Color online) Purity threshold $P_{c}$ given in Equation (21) (green surface) and natural purity limits 1 (blue upper surface) and $\frac{1}{d}$ (red lower surface) as functions of d∈[4,1000] and $d_{m}\in \left[2,25\right]$ such that $d_{m}^{2}\leq d$. Values of purity below the green surface are such that $Q_{1}<Q_{2}$, while values of purity above the green surface yield $Q_{2}<Q_{1}$. It is clear that when $d_{m}^{2}<d$, $P_{c}∼\frac{1}{d}$, meaning that $Q_{2}<Q_{1}$ for most quantum states. On the other hand, when $d_{m}^{2}∼d$ and $Q_{1}<Q_{2}$ almost everywhere in state space.

Two limiting cases are easily studied directly from Equation (21): for a perfectly balanced bipartition ($\sqrt{d}=d_{m}$), one gets $P_{c}=1$ and, since by definition $\frac{1}{d}\leq P\left(\rho \right)\leq 1$, in this case the bound $Q_{1}$ is smaller (and therefore works better) than the bound $Q_{2}$ for any possible quantum state. In the opposite limit, in a strongly unbalanced bipartition, one can roughly approximate ${\left(\sqrt{d}-d_{m}\right)}^{2}∼d$ and, since by definition d≥4, this leads to $P_{c}∼\frac{d\;-\;1}{d^{2}\;+\;d\;-\;1}<\frac{1}{d}$. Taking into account the natural bounds for the purity of a quantum state, this in turn means that in such a limit $Q_{1}>Q_{2}$ for any quantum state or, in other words, our new bound always works better. This behavior can be clearly seen in Figure 1, where the dependence of $P_{c}$ on *d* and $d_{m}$ is shown together with the natural limiting values of P(ρ).

**Figure 2:**
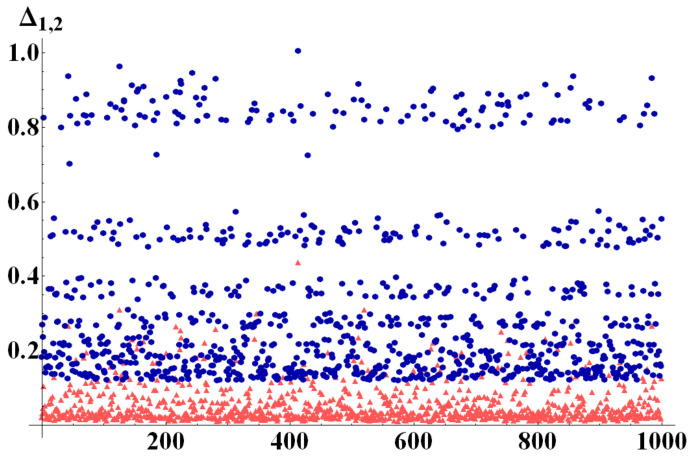
(Color online) $\Delta_{1}$ (light red triangles) and $\Delta_{2}$ (dark blue circles) evaluated for 1000 randomly generated bipartite states, with $d_{m}=d_{M}$ randomly chosen in [2, 10]. For these perfectly balanced bipartite states $\Delta_{1}<\Delta_{2}$ everywhere in state space.

**Figure 3:**
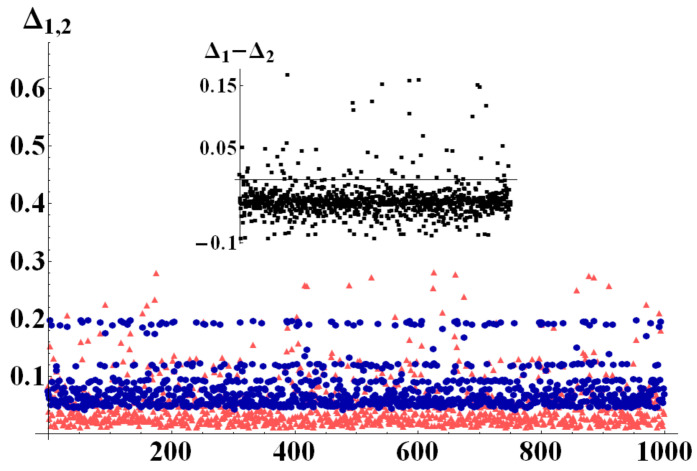
(Color online) $\Delta_{1}$ (light red triangles) and $\Delta_{2}$ (dark blue circles) evaluated for 1000 randomly generated bipartite states, with $d_{M}=d_{m}+60$ and $d_{m}$ randomly chosen in [2, 10]. Since the bipartitions are no longer perfectly balanced, there is a much broader mixing of values of $\Delta_{1}$ and $\Delta_{2}$. In particular, $\Delta_{1}$ has a much wider distribution of values, while $\Delta_{2}$ seems to have a much denser distribution around central values. The inset shows the difference $\Delta_{1}-\Delta_{2}$.

**Figure 4:**
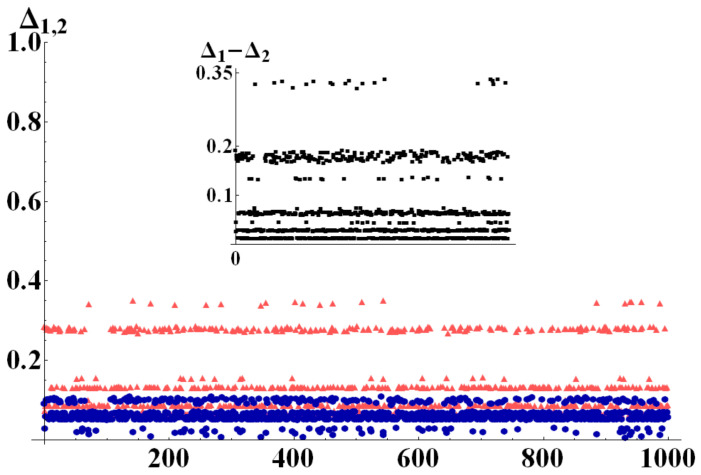
(Color online) $\Delta_{1}$ (light red triangles) and $\Delta_{2}$(dark blue circles) evaluated for 1000 randomly generated bipartite states, with $d_{M}=d_{m}+70$ and $d_{m}$ randomly chosen in [2, 5]. For these strongly unbalanced bipartitions we always detect $\Delta_{1}>\Delta_{2}$. The inset shows the difference $\Delta_{1}-\Delta_{2}$.

To better exemplify this behavior, we report here results of numerical simulations performed with the aid of the QI package for Mathematica [28], by which random quantum states were generated in different dimensions, uniformly distributed according to different metrics. On these states, we tested bounds $Q_{1}$ and $Q_{2}$. Figure 2, Figure 3 and Figure 4 show the differences $\Delta_{i}=Q_{i}-N$ (i=1,2) of the bounds with the negativity of the state, once a bipartition was fixed. In particular, in a first run of simulations (Figure 2) we generated $10^{3}$ perfectly balanced bipartite states (such that $d_{m}=d_{M}=\sqrt{d}$), randomly choosing the dimension of the two subsystems for each quantum state within the range $d_{M}=d_{m}\in \left[2,10\right]$. The results in Figure 2 clearly show that $\Delta_{1}<\Delta_{2}$ for all the analyzed states. The second run of simulations was performed with $d_{m}$ randomly chosen in [2,14] and $d_{M}=d_{m}+60$. In this case, as can be seen in Figure 3 the difference $\Delta_{1}-\Delta_{2}$ has no fixed sign. The two subdimensions are, indeed, such that the critical value of purity $P_{c}$ in Equation (21) is neither extremely close to $\frac{1}{d}$ nor to 1. As can be noticed from the inset of Figure 3 which shows the difference $\Delta_{1}-\Delta_{2}$, however, on the average it is still true that $\Delta_{1}<\Delta_{2}$. The third set of numerical data, finally, was obtained generating $10^{3}$ random states with subdimensions $d_{M}=d_{m}+70$ and $d_{m}$ randomly drawn in [2,5]. In this limit the value of $P_{c}$ is very close to the minimum of purity and we therefore expect $Q_{2}$ to work better than $Q_{1}$ for almost any state. This is indeed confirmed by the simulations shown in Figure 4, in which $\Delta_{2}<\Delta_{1}$. As an example application of our results, consider a single two-level system interacting with a spin system composed of $n_{s}$ spins, each of which lives in a $d_{s}$ dimensional Hilbert space. Therefore, the total system Hilbert space has dimension $d=2{\left(d_{s}\right)}^{n_{s}}$. Let us suppose the spin system is a chain of 10 spin $\frac{1}{2}$ (which is a relatively small system, very far from its thermodynamic limit). The total Hilbert space dimension will then be $d=2^{11}$, and considering the natural bipartition into the two-level system and the spin chain, one has $d_{m}=2$ and $d_{M}=2^{10}$. For such a system, the critical value of purity $P_{c}$ in Equation (21) is

(22) $$P_{c}=\frac{2^{11}-1}{2^{11}{\left(\sqrt{2^{11}}-2\right)}^{2}+2^{11}-1}∼0.000534.$$

The lower value of purity for which bipartite entanglement can survive is, as stated previously, $P_{l}=\frac{1}{d\;-\;1}∼0.00049$. Therefore, for all the total states having purity 0.000534≤P(ρ)≤1, bound $Q_{2}$ in Equation (18) works better than $Q_{1}$. Only for the small fraction of states having an extremely low purity in the range [0.00049,0.000534] bound $Q_{1}$ gives better information than $Q_{2}$. This again shows that, for unbalanced bipartitions (and even in the case of a relatively small number of individual components of the total system), $Q_{2}$ works much better than $Q_{1}$.

## 5. Application to Thermal Entanglement

Of particular interest is the application of the results of this paper to the case of thermal entanglement, where both linear entropy and its link to negativity acquire a much clearer meaning. A recent result [29] indeed shows how the canonical ensemble description of thermal equilibrium stems from the existence of quantum correlations between a system and its thermal bath. In view of this it has been shown that it is possible, with a very small statistical error, to replace the system + bath microcanonical ensemble with a pure state inside the suitable energy shell, still obtaining the appropriate thermal statistics characterizing Gibbs distribution. In this context, then, the linear entropy of the mixed Gibbs state provides a system/bath entanglement measure. The mixedness of a quantum state can originate from the fact that the quantum system S is entangled to another, external system E. If this is the case, the reduced state of the system S is mixed. Moreover, if S and E are maximally entangled, the reduced state of S is maximally mixed. Hence, when one looks at the mixedness of the state of S, one can see it as measuring the amount of entanglement across the S + E bipartition, provided S + E is in a pure state. In this case (and in this case only), our bounds can be seen as resulting from monogamy of quantum correlations: the more S is entangled to E (hence, the more mixed the state of S is), the less subparts of S can create entanglement between them (because they are both already strongly entangled with E). This gives an intuition of the physical origin of our bounds in the case of S being coupled to another system E, and when the two can be considered to be in a pure total state. This is exactly the situation suggested in [29] as being at the origin of Gibbs thermal states. In this sense, thermal mixedness in S can be seen as the result of entanglement in a pure S + E state, E being the thermal environment. Equation (20) can then be viewed as a monogamy relation, describing the competition between two kinds of quantum correlations—internal ones measured by negativity and external ones measured by entropy. On the other hand, it is known that some thermodynamic quantities (e.g., heat capacity or internal energy) can be used as entanglement witnesses [18], and recent works have shown an even closer link between heat capacity and entanglement for particular systems [30,31]. The result of this paper suggests this link might hold very generally. Indeed, in the case of a Gibbs equilibrium state, $P_{E}$ can be given by the expression

(23) $$P_{E}=\sum_{i\neq j}\frac{e^{-\beta E_{i}}e^{-\beta E_{j}}}{Z^{2}}=\sum_{i\neq j}P_{E}^{ij},$$

where $E_{i}$ is the *i*-th energy level of the system and *Z* is its partition function, β being the inverse temperature in units of $k_{B}$. Heat capacity in a finite dimensional system reads

(24) $$C_{V}\equiv \beta^{2}\left(\langle H^{2}\rangle -{\langle H\rangle }^{2}\right)=\beta^{2}\sum_{i\neq j}P_{E}^{ij}\frac{E_{i}-E_{j}}{2}.$$

There is then a similarity between $P_{E}$ and $C_{V}$ as given by Equations (23) and (24), suggesting how a measure of the latter, together with little knowledge about the energy spectrum of the physical system, might supply significant information on the linear entropy of the system and, as a consequence, on its maximal degree of internal bipartite entanglement. This triggers interest in further future investigation on a detailed analysis of the relation between $P_{E}$ and $C_{V}$ which, in turn, might supply us with an easily experimentally measurable entanglement bound as well as highlight how the origin of thermodynamic properties is strongly related to non-classical correlations and monogamy effects. Such a connection, and the usefulness of the bounds derived in the previous sections, can be exemplified with a simple three-qutrit system with a parameter-dependent Hamiltonian

(25) $$H_{l}=\omega J_{z}+\tau J_{1}\cdot J_{2}+{\left(J_{1}\cdot J_{2}\right)}^{2}+kJ_{0}\cdot \left(J_{1}+J_{2}\right),$$

where $J_{i}$ is the spin operator of the *i*-th particle, $J=J_{0}+J_{1}+J_{2}$, and ω,τ,k are real interaction parameters. This effective Hamiltonian operator describes a system consisting of two ultracold atoms (spins labeled as 1 and 2) in a two-well optical lattice and in the Mott insulator phase, where thus the tunneling term in the usual Bose–Hubbard picture is accounted for as a second-order perturbative term, both coupled with a third atom (labeled as 0) via a Heisenberg-like interaction. An external magnetic field is also present, uniformly coupled to the three atoms. Such a system is a generalization of the one studied in [31], where a deep connection between thermal entanglement and heat capacity in parameter space has been shown. Hamiltonian (25) is analytically diagonalizable, thus allowing us to obtain explicit expressions for thermodynamic quantities characterizing the Gibbs equilibrium state of the three-atom system, together with the negativity of the reduced state of the two quadratically coupled spins.

**Figure 5:**
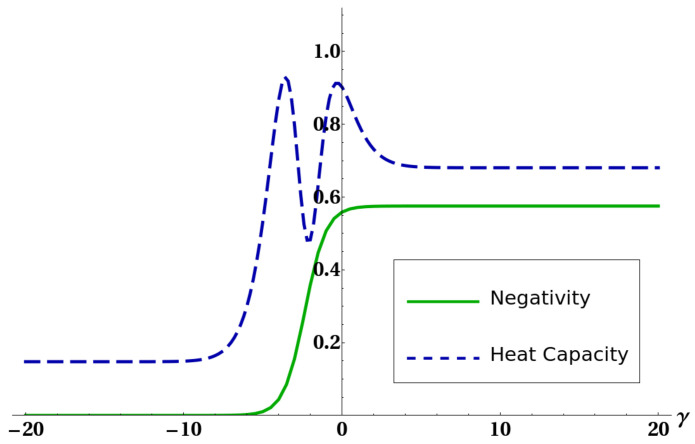
Negativity of the reduced state of the two ultracold atoms(full line) and heat capacity of the system (dashed line) versus quadratic interaction parameter γ. The other parameters were fixed as $k_{B}T=2$, τ=3, and k=1.

**Figure 6:**
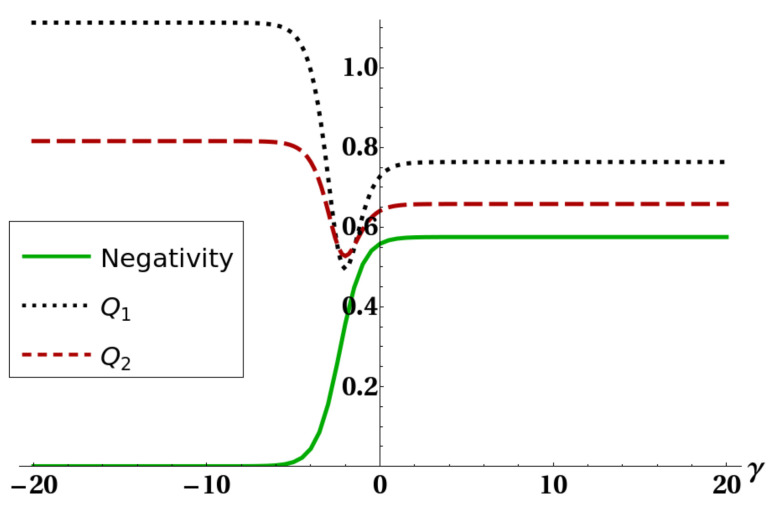
Negativity of the reduced state of the two ultracold atoms (full line), bound $Q_{1}$ (dotted line), and bound $Q_{2}$ (dashed line) versus the quadratic interaction parameter γ. The other parameters were fixed as $k_{B}T=2$, τ=3, and k=1.

**Figure 7:**
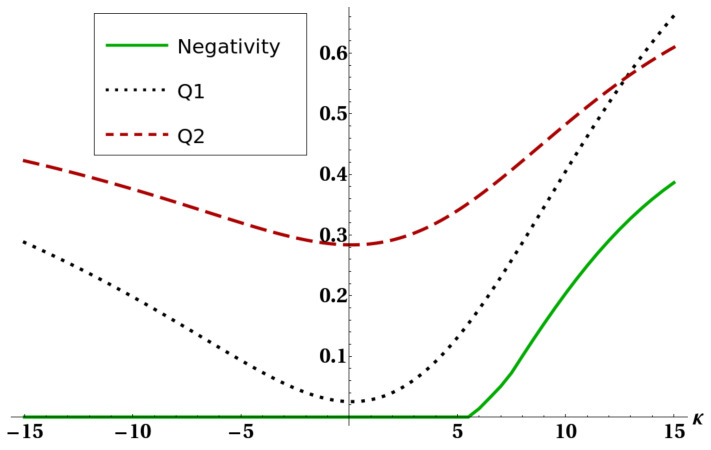
Negativity of the reduced state of the two ultracold atoms (full line), bound $Q_{1}$ (dotted line), and bound $Q_{2}$ (dashed line) versus the Heisenberg interaction parameter *k*. The other parameters were fixed as $k_{B}T=10$, τ=3, and γ=1.

**Figure 8:**
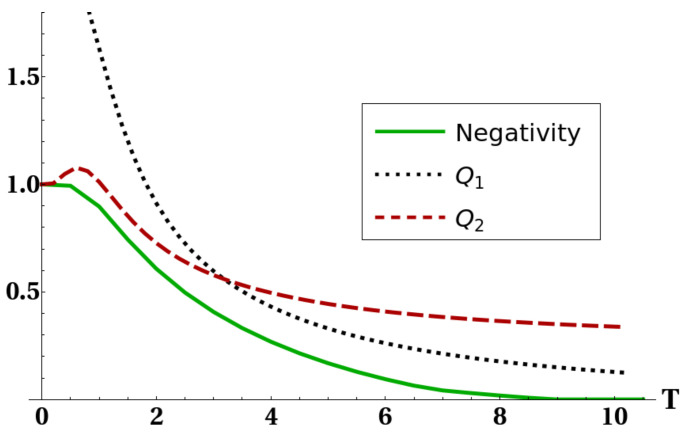
Negativity of reduced state of the two ultracold atoms (full line), bound $Q_{1}$ (dotted line) and bound $Q_{2}$ (dashed line) versus temperature *T* (in units of $k_{B}$). The interaction parameters have been fixed as τ=4, k=5 and γ=1.

The mathematical origin of the connection between heat capacity and negativity was already discussed in [31] and is ultimately due to the presence of level crossing in the low-lying energy eigenvalues of the system. Here we want to show how the existence of the strong connection between purity and negativity, expressed by bound (20), can give some hints for a physical explanation of such an effect, and moreover to exemplify how bound (20) can often supply important information on the amount of thermal entanglement. Figure 5 shows how the connection between thermal entanglement and heat capacity highlighted in [31] is still present despite the interaction with a third atom. Figure 6 and Figure 7 show bounds (5) and (18), together with the negativity of the reduced state of two atoms, versus a certain interaction parameter in the Hamiltonian. Figure 8 finally shows the same quantities versus temperature for fixed Hamiltonian parameters. All energies in the plots are expressed in units of ω. It is worth stressing here that, in all these plots, bound $Q_{3}=\Theta \left(\frac{d\;-\;2}{d\;-\;1}-P_{E}\right)$ is not shown. The reason is that, in order to preserve thermal entanglement, the temperature in our simulations had to be kept at most of the same order of magnitude of spin–spin interactions, and in such a regime $P_{E}$ has not yet crossed the threshold $\frac{d\;-\;2}{d\;-\;1}$ so $Q_{3}$ was constantly equal to one. It is clearly shown in Figure 6 and Figure 8 how bound $Q_{1}$ given in (5) can become, as discussed, larger than 1. In all these cases (except for a small temperature range in Figure 8, however, (18) is still able to sensibly bound negativity. In all the plots shown, and in general every time the bounds (5) and (18) are applied to the particular system analyzed here, one always gets useful information about bipartite entanglement in the form, of course, of an upper bound. However, such a bound gets very close to zero in some particular cases (e.g., Figure 7 and Figure 8), strongly restricting the allowed range of values for the negativity. It is then shown that (20) is able to produce non-trivial results. It is worth noting that in Figure 5 there are ranges of the parameter γ where the negativity and the heat capacity exhibit simultaneous plateaus. This fact, also previously shown and commented in [31], in view of Equation (20) and the strong link between heat capacity and the mixedness $P_{E}$ of a quantum state, legitimizes the deduction that in the parameter regions of very low negativity the heat capacity may be assumed as almost constant.

## 6. Conclusions

In this paper we derived a bound on the degree of information storable as bipartite quantum entanglement within an open *d*-dimensional quantum system in terms of its linear entropy. Our result is quite general, holding for arbitrary bipartitions of an also arbitrary system. Indeed, our work concerns any bipartite quantum system of finite dimension. Examples may include coupled quantum dots, interacting atoms or molecules, different degrees of freedom of photons, or superconducting circuits, but this is a very limited list of examples of typical experimental realization of quantum systems that are easy to manipulate. The same is true for the states considered: our results apply to any quantum state of finite-sized bipartite quantum systems. As a matter of fact, as shown in this paper, all we need to specify is the purity of such states, which is well-defined for any quantum state. We emphasize that our result is experimentally appreciable in view of quite recently proposed protocols aimed at measuring the purity of a state of a quantum system. Inspired by the seminal paper of Popescu, Short, and Winter [29], our conclusions highlight the interplay between quantum entanglement inside a thermalized system and its physical properties. Our results are of interest not only for quantum information researchers, but also for the growing cross-community of theoreticians and experimentalists investigating the subtle underlying link between quantum features and thermodynamics.

## Appendix A

**Proof** **of Lemma 1.** Let us first notice that both B(t) and 1−A(t)−B(t) go to zero when the state τ(t) is pure. Indeed, $A+B=\mathrm{Tr}\tau^{2}$ equals the purity, while *B* by construction vanishes when τ is pure (see Equations (9)–(13)). Notice further that both A(t) and B(t) are quadratic in $\nu_{\beta }\left(t\right)$ and $\mu_{\alpha }\left(t\right)$ and then the statement of Lemma 1 is independent of their sign. Such a statement can be rewritten as

(A1) $$\frac{1-A-B}{d}-B=l\left(A,B\right)\geq 0.$$

The function l(A,B) at the extremal points of its domain (corresponding to a pure and a maximally mixed state) satisfies (A1). For a maximally mixed state, calling $n_{\alpha }$ $\left(n_{\beta }\right)$ the number of α- (β-)class eigenvalues $\left(n_{\alpha }+n_{\beta }\right)$, one gets $A=\frac{n_{\alpha }}{d^{2}}$ and $B=\frac{n_{\beta }}{d^{2}}$ and thus $l=\frac{n_{\alpha \;-1}}{d^{2}}\geq 0$ since $n_{\alpha }\geq 1$. Let us now express l(A,B) as

(A2) $$h\left(\left\{\mu_{\alpha }\right\},\left\{\nu_{\beta }\right\}\right)=\frac{1}{d}\left(1-\sum_{\alpha }^{n_{\alpha }}\mu_{\alpha }^{2}-\sum_{\beta }^{n_{\beta }}\nu_{\beta }^{2}\right)-\sum_{\beta }^{n_{\beta }}\nu_{\beta }^{2}.$$

We can address the investigation on internal points using the Lagrange multiplier method, taking into account the trace condition $\sum_{\alpha }^{n_{\alpha }}\mu_{\alpha }^{2}+\sum_{\beta }^{n_{\beta }}\nu_{\beta }^{2}=1$. From this method, only one stationary point results, characterized by values of $\nu_{\beta }$ and $\mu_{\alpha }$ such that the corresponding state is mixed. It is straightforward to check that at this point the function (A2) is positive. We then deduce that l(A,B)≥0, from which Lemma 1 directly follows. Finally, the range δ of validity of Lemma 1 is given by the requirement $q_{R}\left(t\right)\geq q_{i\neq R}\left(t\right)$, such a property being necessary for the sensible definition of the reference pure state $\sigma_{R}$ which guarantees, in turn, that B(t) vanishes on pure states. □
